# Random-effects psychophysics for studying individual differences in perception and cognition

**DOI:** 10.3758/s13423-026-02890-y

**Published:** 2026-04-20

**Authors:** Jeffrey N. Rouder, Mahbod Mehrvarz

**Affiliations:** Department of Cognitive Science, University of California, Irvine, CA 92697 USA

**Keywords:** Psychophysics, Hierarchical models, Random effects, Individual differences, Perception

## Abstract

In modern psychological science, many researchers use psychophysical tasks to study individual differences in perception, attention, aging, and personality. Psychophysics, however, traditionally uses designs with a great many trials and few individuals. These designs are inappropriate for individual-difference studies. Here, we develop a random-intercept psychophysics that jointly models variation across trials and individuals within a Bayesian hierarchical framework. We show that the model is ideal for measuring thresholds in small-trial designs. Because the model jointly accounts for variation across trials and individuals, it provides an assessment of correlation across tasks without the pernicious attenuation caused by trial noise. The resulting correlations are accompanied by measures of uncertainty that reflect both the number of trials per individual and the number of individuals. Because the framework is Bayesian, it is flexible, and we leverage this flexibility in two ways: First, we place factor models on the thresholds themselves, demonstrating how the structure of individual differences across a battery of tasks may be assessed. Second, we develop a custom-tailored psychophysical model for assessing whether stimulation is subliminal or superliminal. The threshold divides at chance-performance form above-chance performance, and the approach serves as a principled approach for assessing truly subliminal priming.

Psychophysics is the study of how people represent and process sensation (Falmagne, 1990; Stevens, 1957). A modal research investigation proceeds as follows: The researcher first measures an individual’s performance across a set of critical conditions. With these measurements in hand, the researcher derives a mathematical relationship for the change of performance across the conditions. This relationship serves as a rich, to-be-explained phenomenon for subsequent theories. Examples of successes using this modality include the Fechner–Weber law (Dehaene, 2003; Fechner, 1860; Masin et al., 2009), critical band theory in audition (Fletcher & Munson, 1933; Greenwood, 1961), and the opponent theory of color perception (Hurvich & Jameson, 1954), among many others.

Unfortunately, psychophysics and psychophysical techniques have a limited impact in today’s psychological sciences. We say this not to provoke or to disparage, but to propose a *win-win* set of statistical developments and design considerations to increase impact. The limited impact comes about, ironically, because psychophysical researchers prioritize high-precision measurements of performance. They do so because this high degree of precision is needed to discover mathematical relationships across conditions. The main method of gaining this precision is to require that individuals perform a great many trials, the completion of which takes several hours and may span weeks of time in the lab. This commitment to large trial sizes comes with an obvious drawback—it is feasible to run only a handful of individuals. And herein lies the heart of the problem. With a few individuals, the variability across the population cannot be studied. The first implication is that any generalization to a population is made informally without recourse to the variability across individuals. The second implication is that individual differences cannot be studied, and that their correlates remain outside the scope of study. The third and perhaps most important implication is that most cognitive psychologists will not run experiments with so many trials and so few individuals, meaning that psychophysical studies are now quite rare. The goal here is to provide hierarchical models for psychophysical tasks so performance can be measured with fewer trials, and variation across individuals may be studied.

In modern literature, psychophysics may rarely be an object of study, but psychophysical tasks are nonetheless used in cognitive, social, and personality studies. In these psychophysics-adjacent fields, perception is studied to the degree it plays an important role in attention, control, aging, emotional well-being, and intelligence. Here are two examples we use throughout:

## Perception and intelligence

One of the key questions outside of psychophysics is how differences in perception across individuals affect performance in abstract tasks such as reading and reasoning. Galton (1883) famously hypothesized that sensory abilities were correlated with intelligence, and modern tests of this proposition include Jastrzȩbski et al. (2021). and Tsukahara et al. (2020). The modern claim is that sensory discrimination is related to IQ, though it may be mediated by attentional control. Perhaps the most salient relationship is between visual backwards masking and IQ (Deary & Stough, 1996). In a backwards-masking task, a target stimulus is briefly flashed. Then, after a subsequent delay, a masking stimulus is presented. The delay, the *interstimulus interval* (ISI), may be interpreted as measuring how fast individuals can read out target information from iconic memory to a more durable store before the mask appears (Sperling, 1960). Backwards masking correlates about .5 with WAIS (Grudnik & Kranzler, 2001). The fact that this low-level speed of iconic readout is correlated so strongly with abstract tasks in WAIS is surprising. The phenomenon highlights the salience of perception for higher-order cognitive tasks.

## Priming without awareness

Another example where psychophysics plays a critical role is the exploration of subconscious processing. The common claim is that there are stimuli that are too faint to trigger awareness that nonetheless affect behavior (a selective list includes Arndt et al., 2001; Bar & Biederman, 1998; Dehaene et al., 2003; Elgendi et al., 2018; Greenwald et al., 1996; Vorberg et al., 2003). Though this claim is popular, it remains controversial (Dosher, 1998; Newell & Shanks, 2014; Pratte & Rouder, 2009). Studying the relationship between stimulus strength and awareness is a classical psychophysics question (Fechner, 1860), yet psychophysical theory and methods are not typically leveraged in these studies (cf., Dagenbach et al., 1989).

In most psychology experiments, there are (at least) two sources of variation. One is *within-person* variation—how performance for a certain individual varies across the trials. The other is *between-person* variation—how people vary and, in particular, how they vary across conditions or tasks. Psychophysical methods address within-person variation without consideration of between-person variation. Yet, in both the applications highlighted above, between-person variability plays a critical role. In the backwards-masking application, two scores are tabulated per participant—a backwards-masking score and an IQ score—and the correlation across these two is the phenomenon of interest. Likewise, in subliminal priming, most researchers use significance testing to show a lack of awareness in a detection task while showing priming in a priming task. These significance tests leverage between-person variation.

The hierarchical approach advocated here addresses both within-person and between-person variation simultaneously. Addressing both sources has direct statistical benefits. First, thresholds may be estimated better with fewer trials. Second, incorporating between-person variation provides for better articulation with psychophysics-adjacent questions. In the priming-without-awareness example, we can define individual at-chance thresholds and use *bona fide* psychophysical methods to estimate these thresholds and our uncertainty in them (Morey et al., 2018). In the backwards masking example, we can better estimate individual backwards-masking abilities. With these improvements, we are better positioned to understand how much attenuation in the correlation is due to measurement error. Moreover, we are better positioned to explore why this surprising phenomenon occurs by studying how this relationship between threshold and IQ varies across factors known to affect iconic memory and read-out speeds (e.g., Pratte, 2018; Sperling, 1967). Centering individual variation increases the impact and relevance of psychophysics in psychological sciences.

Hierarchical models are comprised of two levels. At the first level, called the *data level*, trial-by-trial responses are modeled with individual-level parameters, such as their threshold. At the next level, the *latent level*, these latent individual parameters are treated as random effects; they are assumed to be from a common or population-level distribution. This last assumption places constraints on individuals—they cannot be arbitrarily different from the population. We call this approach “random-effects psychophysics” to highlight this main feature. Individuals’ true effects are treated as random, and the distribution of these random effects reflects underlying substantive structure about the domain at hand.^1^ At a minimum, this structure may be leveraged to improve estimation of individuals’ thresholds; but, perhaps as importantly, the structure itself becomes a target of study. Its form—how people vary across tasks and conditions—is a scientific question unto itself.

Hierarchical approaches have been proposed in experimental psychology for at least twenty years (see, for example, Peruggia et al., 2002). Rouder and Lu (2005) provides an early treatment of response accuracy data with a hierarchical signal-detection account. Hierarchical approaches to estimating psychometric functions in psychophysical contexts have been developed by Balestrucci et al. (2022), Houpt and Bittner (2018), Kim et al. (2014), and Prins (2024). It is only recently, however, that hierarchical models have been used to center individual differences across different experimental tasks. Matzke et al. (2017) provides a hierarchical approach for measuring the correlation between two tasks in the presence of trial noise, and Rouder et al. (2023) provide a richer set of models for exploring full correlation matrices across many tasks in high-noise settings. Vandekerckhove (2014), Turner et al. (2017) and Stevenson et al. (2024) provide hierarchical models that merge process-models—the diffusion model in their cases—with factor models of individual differences. Mehrvarz et al. (2026) developed a hierarchical factor-model to uncover a general susceptibility to illusions factor that governs individual differences across various illusion tasks.

The models developed here follow in this hierarchical-model tradition. The probability of a correct response on any trial is modeled as arising from psychophysical models with threshold parameters, and individual differences in thresholds across tasks, conditions, and subgroups are governed by structured models such as factor models. Perhaps the key to the models presented here is simplicity. We model individuals as varying in a single way or on a single dimension per task (cf., Kujala & Lukka, 2006; Prins, 2024). This simplified version is geared for psychophysical-adjacent fields where there are fewer trials per individual in a task and the data lack the resolution to estimate multiple dimensions of individual variation within a task.

In Section “Better estimation through hierarchical models”, we review how hierarchical models provide for more accurate estimation for individual’s parameters and for structural parameters that describe the variation across individuals. This review is fairly generic, and readers familiar with regularization in statistics may skip it without loss. Then, with this benefit in mind, we introduce random-effects psychophysics for a single task in Section “Random-effects psychophysics for a single task”. The key goal is the measurement of individual thresholds. The ensuing Bayesian analysis is computationally tractable, provides the needed regularization, and yields credible assessments of uncertainty. Two synthetic examples—one with the method of constant stimuli and another with an adaptive staircase (Levitt, 1971)—show how random-effect psychophysics improves analysis. This demonstration is followed by a real-world example from Tsukahara et al. (2020) where individuals’ ability to discriminate the area among similar circles is measured. We show here how the hierarchical model allows for superior estimation when there are few trials per individual. In Section “The correlation between two tasks”, we introduce a multivariate approach for measuring relations across tasks and conditions. We explore the bivariate correlation across line-length and visual-area discrimination in the Tsukahara et al. data set and find the model greatly disattenuates correlation coefficients, revealing hidden relations among the tasks. The model framework is extended in Section “Factor models” to incorporate factor models across individuals and tasks. In Section “Thresholds for unconscious processing”, we address thresholds for subliminal priming. Conventional thresholds do not separate subliminal from superliminal performance. For example, in a two-choice task with floor performance at .5, subliminal performance is at this floor while superliminal performance is above this floor. Conventional thresholds typically aim for .707 or .75, which is well above the subliminal divide. We present models that estimate a threshold floor level of performance. These models can then be used to assess subliminal priming, where subliminal refers to the effect of stimuli presented below the at-chance threshold. Section “Model misspecification” is about the assessment and implications of model misspecification. We provide a graphical approach to exploring misspecification and advise on interpreting and mitigating misspecification issues.

**Fig. 1:**
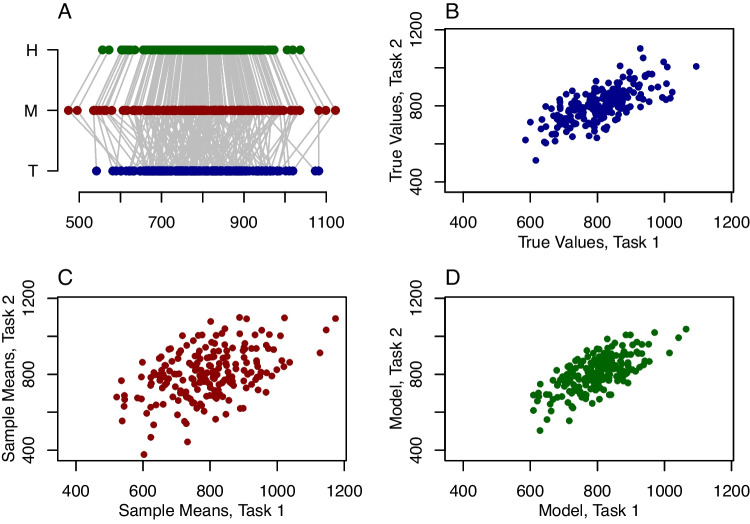
Examples of regularization. **A** Regularization in a single task is a shrinkage toward the grand mean. The *bottom dots*, labeled ‘*T*’ are true values for individuals; sample means across individuals, labeled ‘*M*’, are perturbed by noise, leading to more variability than true values. Hierarchical estimates, labeled ‘*H*’, provide for better estimation by shrinkage toward the grand mean. **B**–**D** Regularization across two tasks as shrinkage toward a regression line. **B** True scatter plot across individuals. **C** Sample means are perturbed by noise and lead to the well-known attenuation of correlation. **D** Shrinkage toward a regression line corrects the attenuation and is known disattenuation

## Better estimation through hierarchical models

A key concept in estimation is accuracy—estimates are observed quantities that should accurately estimate an unknown. The question then is how to make the estimation maximally accurate. To serve this goal, there has been a pronounced move in statistics towards *regularized* estimation. Regularization describes how estimation should be tempered by readily available external information to maximize accuracy. Examples of common regularized estimates include ridge regression, lasso estimation, estimation within hierarchical models, and empirical Bayes estimation (Casella & Berger, 2002). An example is helpful; suppose we wish to estimate the true speed of each individual in a set. One approach is to estimate each individual’s speed using only that individual’s data, perhaps by taking the mean response times across that individual’s trials. In regularized approaches, the individual’s sample mean is combined with information from the group of individuals such that the estimate is informed by both person-specific and group-level data.

Stein (1956) first showed that estimation improves when external information is *appropriately* incorporated. The implications of this proof are stunning—for example, the estimates of the number of cattle in Montana, the price of tea in China, and the temperature of the Ganges River were improved on the whole if each was *appropriately* allowed to influence the other. The key here is the word appropriately, and what is appropriate will become apparent in the following examples. Moreover, the nature of the external information is relevant too. Even though adding any is helpful, adding more relevant information is even more helpful. The take-home message is that regularized estimators are more accurate than conventional sample statistics such as the sample mean. And that is why they dominate modern statistics.

Some experimental psychologists are already familiar with regularization and routinely use modern estimators such as LASSO or hierarchical models to improve the accuracy of their estimates. For many others, however, regularization may not yet be part of their toolkit, so a brief review is useful. In this section, we show how hierarchical-model analysis, as a form of regularization, can yield more accurate estimates. The ideas themselves are not new; they are standard in quantitative psychology, and our discussion follows closely that of Efron and Morris (1977). They may, however, be new in this context, and our hope is that they help shape how you think about analysis.

**Fig. 2:**
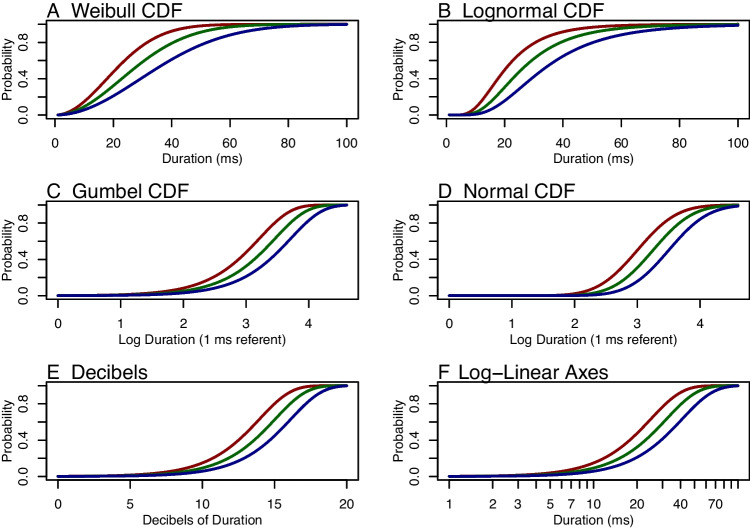
Psychometric functions on intensity and log-intensity scales

### Regularization within a task

Let’s start with 200 people each performing a response-time task. Figure 1A shows three rows. The bottom row, denoted “T” is the true values for each of these 200 people, and it is what we wish to estimate. True values are the values we would obtain if we could collect a large number of trials from each individual. In these synthetic data, people are quite variable; their true scores range from 550 ms to 1100 ms. In any experiment, we observe a sample where each observation reflects this true value perturbed by noise. The second row, denoted “M”, shows the sample mean across ten such observations per individual. By and large, these observations follow the true values, but because of trial noise, they are not exactly at the true values nor in perfect correspondence. The sample means subtend a greater range than the true values, which is a natural consequence of trial noise.

The upper row, denoted “H”, shows regularized estimates from a hierarchical model. The estimate is a weighted average of two parts: one part comes from the specific individual’s data; another part, the regularization, comes from everyone else. The part from the individual is the sample mean, and it is heavily weighted in the example. The part from everybody else is the overall mean across all people, and it is lightly weighted. The effect is to shrink or pull the estimates toward the overall mean. The shrinking accounts for the fact that the range of sample means must be greater than the range of true values. The shrinkage results in increased accuracy. In the example, the sample means miss their corresponding true values by 71 ms on average. The hierarchical estimators miss by only 60 ms on average, a reduction of 16%.

### Regularization across tasks and the estimation of correlations

It is popular to estimate the correlation of individuals’ performance across tasks. One problem in this endeavor, as pointed out by Spearman (1904), is that the usual estimate of correlation is attenuated by measurement error. How this attenuation happens is shown in Fig. 1B and C. Panel B shows true values for 200 people in two tasks. There is a large degree of correlation—people who have high true values in one task have high true values in the other. Sample means are shown in panel C. Sample means are true values perturbed by noise, and the perturbation can be in any direction. The result is a smearing of the structure; the observed correlation is too small in magnitude. The degree of attenuation is a function of the number of trials per individual—the fewer trials, the greater the attenuation.

Regularization is useful for disattenuating correlations (Haines et al., 2020; Matzke et al., 2017; Rouder & Haaf, 2019). In the previous case (Fig. 1A), regularization shrank individual estimates toward a single overall mean. In the bivariate case, a hierarchical model shrinks each pair of scores toward the regression line relating the two variables, as shown in Fig. 1D. The slope and intercept of this line are not assumed, but estimated from the data along with the true values. The result is a disattenuated correlation that better reflects the underlying association.

Researchers can better understand the structure in data by using hierarchical models that simultaneously account for variation within trials and across people. Hierarchical models assume that differences among people are constrained by structure at the population level. This constraint is not only reasonable but also directly provides for a better understanding of individuals themselves as well as the population structure that governs them.

## Random-effects psychophysics for a single task

A hierarchical psychophysics model has two levels of specification: a *data level* where trial-by-trial responses are modeled, and a *latent level* where individual latent parameters are modeled.

### Specification

#### Data level model

Let $d$ be the *intensity* of a stimulus. Intensity has a lower limit of zero and is measured in physical units. In audition, it may be the sound pressure measured in pascals (Pa), in vision it may be the luminance measured in candelas-per-square-meter (cd/m$^2$), and in backward masking, it may the duration of the interstimulus interval measured in milliseconds (ms). Let $p()$ be the psychometric function that maps from intensity into accuracy. Figure 2A shows an example of three such functions, one for each of three hypothetical individuals. In the figure, the intensity variable is duration. Key here is that (i) intensities are nonnegative, and (ii) the function is always increasing, that is, people perform better the greater the stimulus intensity. Given these properties, it is appropriate to use the cumulative distribution function (CDF) of a distribution that has support on the nonnegative numbers, and the curves in Fig. 2A are the CDF of the Weibull distribution.

The psychometric function for the Weibull is

$$ \text{ Weibull } \text{ CDF }: \quad p(d,\tau _i,\beta ) = 1-\exp \left( -\left( \frac{d}{\tau _i}\right) ^{\beta }\right) , \quad i=1,\ldots ,I. $$

First, the left-hand side. The first argument is the intensity $d$, which is straightforward. The next argument is $\tau _i$, which is an individual parameter describing how much intensity is needed for the $i$th individual to reach a criterial level of performance. The parameter has the same physical units of intensity, say ms or cd/m$^2$. This parameter $\tau _i$ describes how *bad* a person is at the task—higher values indicate that greater intensities are needed for the criterial level. Because $\tau _i$ describes the effects of an individual, there are as many of these parameters as there are individuals. The parameter $\beta$ is a shape parameter and controls how steep the upper part of the curve is. There is a single value of $\beta$ that holds for all individuals. Hence, the difference between individuals’ psychometric curves is captured only by a single parameter, $\tau$. The use of a one-parameter family is common (e.g., Watson & Pelli, 1983) but not universal, as some researchers estimate a separate shape parameter per individual Prins (2024). On the right-hand side, the critical relation is that intensity $d$ and parameter $\tau _i$ enter as a ratio, $d/\tau _i$. Parameter $\tau _i$ is the *intensity characteristic* for the $i$th individual.

Another choice for the psychometric function is the lognormal:

$$ \text{ Lognormal }: \quad p(d,\tau _i,\beta )= \Phi \left[ \log \left( \left( \frac{d}{\tau _i}\right) ^{\beta }\right) \right] ,\quad i=1,\ldots ,I. $$

Figure 2B shows three curves; parameters $\tau _i$ and $\beta$, scale and shape parameters, play the same role here as with the Weibull CDF. A general formulation is

$$ \text{ General }: \quad p(d,\tau _i,\beta )= F_+\left( \frac{d}{\tau _i},\boldsymbol{\beta }\right) ,\quad i=1,\ldots ,I, $$

where $F_+$ is a CDF on positive intensities, $\tau _i$ is an intensity characteristic that describes individual effects, and $\boldsymbol{\beta }$ is collection of other parameters. The main constraint here is that psychometric functions vary across individuals in one way, namely in scale.

Psychophysical models are often expressed in terms of the logarithm of intensity rather than intensity itself. Let $s = \log (d)$ be the strength of the stimulus, where strength is the logarithm of intensity. Figure 2C–D shows the psychophysical functions as a function of stimulus strength rather than stimulus intensity. Let $p^*$ be the function that maps strength into accuracy. Consider the Weibull model on intensity. The corresponding psychometric function on strength is

$$ \text{ Gumbel }: \quad p^*(s,\theta _i,\beta ) =1-\exp (-\exp [\beta (s-\theta _i)]),\quad i=1,\ldots ,I. $$

Here, $\theta _i=\log (\tau _i)$ describes how much strength is needed for a level of performance; it is the strength characteristic for the $i$th person. And, as can be seen in Fig. 2C, the effect of the individual is to shift or translate the function. This function is the CDF of a Gumbel distribution (Johnson et al., 1994). As an aside, the above Gumbel CDF is often used in practice but mistakenly referred to as a Weibull CDF (King-Smith et al., 1994; Nachmias, 1981; e.g., Watson & Pelli, 1983). The Gumbel distribution has mass on all real numbers and is appropriate for strength or decibel measures; the Weibull distribution has mass on only the positive real numbers and is appropriate for intensity.

Consider the previous lognormal model on intensity. The corresponding psychometric function on strength is:

$$ \text{ Normal }: \quad p^*(s,\theta _i,\beta ) =\Phi \left[ \beta (s-\theta _i)\right] ,\quad i=1,\ldots ,I. $$

Moreover, a general psychometric formulation of strength is:

$$ \text{ General }: \quad p^*(s,\theta _i,\boldsymbol{\beta })= F(s-\theta _i,\boldsymbol{\beta }),\quad i=1,\ldots ,I, $$

where $F$ is a CDF on real-valued strengths, $\theta _i$ is a location parameter that describes individual effects, and $\boldsymbol{\beta }$ is collection of other parameters. The main constraint here is that psychometric functions vary across individuals in one way, namely in location (or, equivalently, in shift or in translation). In the parlance of regression models, these models are random intercept models on stimulus strength. The slopes are constant across all individuals.

#### Units of strength

It is helpful for some readers to take a brief detour about the units of strength or log intensity. One might be tempted to think the log scale on duration is measured in log-milliseconds; the log scale on sound pressure is log-millipascals; and the log scale on illuminance is log-candelas-per-square-meter. None of these units, however, exists. There is no log-millisecond unit. One can certainly take the log of a pure number without units, such as $\log (10)$, but one cannot take the log of a quantity that has physical units.^2^ When we take the log of a physical quantity, we implicitly assume there is a referent quantity to cancel units. For example, let $d$ be a duration in milliseconds and let $s=\log (d)$ be a strength. The equation $s=\log (d)$ is shorthand for $s=\log \left( \frac{d}{d_r}\right)$ where both $d$ and $d_r$ are in milliseconds so that the units cancel. The amount $d_r$ ms is the referent, and, implicitly, it is set to $d_r = 1$ ms. Hence $s$ is a pure number, and $\exp (s)$ is the ratio $d/d_r$. So, if $d$ is 10 ms, then the $d/d_r$ is 10, a pure number, and $s=\log (10)=2.3$ is also a pure number describing how many multiples of $s$ is $d$ from $d_r$. Readers often need not know the exact value for the referent (for example, the little-known referent for sound pressure is 20 micropascals), but they do need to know there is an implicit referent. Strengths are relative measures; they are logs of pure-number multipliers of the referent.

In Fig. 2C-D, the term “Log Duration (1 ms referent)” is used as a shorthand for the log of duration after dividing by the referent. In many psychophysical studies, especially those in hearing and audition, the term “decibel” or dB is used. Decibels are a strength measure given by $s'=10\times \log _{10}(d/d_r)$, where $s'$ is the strength in decibels. Figure 2E shows the Gumbel CDF as a function of stimulus strength measured in decibels.

One way to represent the relationship between intensity and strength is to use an older technique from the last century, when scientists drew graphs by hand. Most of us are familiar with the common *linear-linear* graph paper where equal physical increments on the paper correspond to equal increments of the values on the axes. There are alternative graph papers, and the appropriate alternative here is log-linear graph paper. Here, the x-axis is on a logarithmic scale where equal physical increments on the paper correspond to equal increments of the power of the values on the *x*-axis. Figure 2F shows the log-linear spacing—equal physical spacing on the *x*-axis corresponds to changes of a power of 10. The advantage here is that the log-values may be plotted but labeled with the physical unit, ms in this case. Moreover, the referent need not be stated as it is included in the axis values. Log-linear graphs—labeled as function of intensity as in Fig. 2F—are convenient.

**Latent-level model.** The next step is to place a model on $\theta _i$, the strength characteristic for the $i$th individual. For a single task, a normal model is useful:

$$ \theta _i \sim \text{ Normal }(\mu ,\sigma ^2), $$

where $\mu$ is a population-level mean and $\sigma ^2$ is a population level variance. Because $\mu$ and $\sigma ^2$ are parameters, the model is hierarchical and leads to regularization. The key parameter here is $\sigma ^2$, which serves as the size of the signal in individual differences. The larger $\sigma ^2$, the more different people are, and the fewer trials it will take to discriminate them. The three curves in each panel of Fig. 2 denote different individuals at the 16th, 50th, and 84th percentiles. For the normal CDF of strength, Panel D, the curves correspond to $\sigma =.25$ on the natural log scale or 1.08 dB on the decibel scale. To our knowledge, these curves are fairly representative of individual differences in backwards masking.

#### Complete model

The complete model may be written as follows: Let $Y_{ij}=0,1$ be the performance for the $i$th individual ($i=1,\ldots ,I$) on the $j$th trial ($j=1,\ldots ,J_i$) where $0$ and $1$ denote error and correct responses, respectively. Let $s_{ij}$ denote the strength of the stimulus on the $j$th trial for the $i$th individual. The general model, that is, one without the functional form specified, is:

$$\begin{aligned} Y_{ij} \mid \theta _i\sim &  \text{ Bernoulli }[p^*(s_{ij},\theta _i,\beta )],\\ \theta _i\sim &  \text{ Normal }(\mu ,\sigma ^2). \end{aligned}$$

One needs to pick a functional form for $p^*$, and the CDF of the normal is convenient in analysis:

$$\begin{aligned} \mathcal {M}_1: \quad Y_{ij} \mid \theta _i\sim &  \text{ Bernoulli }\left( \Phi \left[ \beta (s_{ij}-\theta _i)\right] \right) ,\\ \theta _i\sim &  \text{ Normal }(\mu ,\sigma ^2). \end{aligned}$$

Alternatively, the model may be expressed as a function of intensity as:

$$\begin{aligned} \mathcal {M}_1: \quad Y_{ij} \mid \theta _i\sim &  \text{ Bernoulli }\left( \Phi \left[ \beta \left( \log \left( \frac{d_{ij}}{d_r}\right) -\theta _i\right) \right] \right) ,\\ \theta _i\sim &  \text{ Normal }(\mu ,\sigma ^2). \end{aligned}$$

### Analysis

#### Conditional independence

The main methodological hurdle in analysis is the lack of independence among trials in adaptive staircase designs. In these designs, the intensity is decreased if the individual is performing well and increased if the individual is performing poorly. Performance data, therefore, have trial-to-trial dependency by design. This dependency is a negative correlation between the current response and the previous one for the obvious reason that performing incorrectly leads to more intense stimuli and performing correctly may lead to less intense ones. The common solution is to make a mild *conditional independence* assumption (e.g., Watson & Pelli, 1983). Here is how it works: Let $Y_{ij}$ be the performance on the current ($j$th) trial for the $i$th individual, and let $\boldsymbol{R}_{i,j}=(Y_{i1},Y_{i2},\ldots ,Y_{i,j-1})$ be a sequence of all previous responses, and let $\boldsymbol{D}_{ij}=(d_{i1},d_{i2},\ldots ,d_{ij})$ be a sequence of all previous durations and the current one. The conditional independence condition is that the current response depends only on the current duration, that is, $\Pr (Y_{ij} \mid \boldsymbol{R}_{i,j},\boldsymbol{D}_{ij}) = \Pr (Y_{ij} \mid d_{ij}).$ The influence of the previous trials is in setting the current duration, and conditional on this duration, the previous trials have no additional influence. Indeed, this mild conditional independence assumption is implicit in Model $\mathcal {M}_1$ as $Y_{ij}$ depends on $s_{ij}=\log (d_{ij})$ without further dependence on previous responses.

#### Bayesian analysis

Analysis of the model may be done in the Bayesian framework. In this framework, priors are needed on $\beta$, $\mu$ and $\sigma ^2$. Conjugate priors with weak information are convenient:

$$ \begin{aligned} \beta&\sim \text{ Gamma }(q_\beta ,r_\beta )\\ \mu&\sim \text{ Normal }(m,v)\\ \sigma ^2&\sim \text{ Inverse } \text{ Gamma }(q_{\sigma ^2},s_{\sigma ^2}) \end{aligned} $$

In the gamma and inverse gamma distributions, $q$ is a shape parameter, and $r_\beta$ and $s_{\sigma ^2}$ are rate and scale parameters, respectively. In the normal distribution, $m$ and $v$ are the mean and variance parameters, respectively. In this context, where the data are binary, the priors may be set broadly. For example, in backward masking, the intensity characteristic $\tau$ typically ranges from 10 ms to 100 ms, and the corresponding strength characteristic $\theta$ ranges from 2 to 5. We use prior settings $m=0$ and $v=100$, a distribution that covers all possible strength characteristics. Prior settings for $\beta$ are $q_\beta =r_\beta =.5$ and, likewise, the prior settings for $\sigma ^2$ are $q_{\sigma ^2}=s_{\sigma ^2}=.5$, which are exceedingly broad as well (Liang et al., 2008; Rouder et al., 2012).

#### Computations

Analysis can proceed by one of two ways. The first way is the old-and-hard way. The analyst derives expressions for conditional posterior distributions using Bayes’ rule, figures out how to sample from these posteriors, and marginalizes using Markov chain Monte Carlo (MCMC) integration (Gelfand & Smith, 1990). If one wishes to analyze the model this old and hard way, the crux move is an elegant data augmentation step to account for the probit transformed binomial data from Albert and Chib (1993). Rouder and Lu (2005) provide a tutorial on this old and hard way, and includes the Albert and Chib data augmentation step.

The second way to analyze the model—the new and easy way—is to use one of the two brilliant off-the-shelf, general-purpose, Bayesian analysis packages: Stan (Carpenter et al., 2017) and JAGS (Plummer, 2003), These well-supported and well-documented packages require model specification as input. Model specification inputs are made in a notation similar to the formal mathematical notation used above. These packages are powerful and capable applications that often work better in real-world contexts than hand-coded boutique solutions from the old-and-hard way. We heartily recommend them, so much so that we recommend teaching them to young scholars as part of an experimental psychology methods curriculum. JAGS is used here rather than Stan because JAGS is a tad more intuitive for those learning Bayesian analysis. JAGS code for all models is included in the working Rmarkdown version of this paper at https://osf.io/sakt5/files/osfstorage.

### Application I: Synthetic data with the method of constant stimuli

Consider an example where each of 50 individuals performs 60 trials in a backwards-masking task. Each individual’s probability of correct response follows a lognormal psychometric function (Fig. 2B and D). What varied across individuals are the intensity characteristics $\tau _i$ (Fig. 2B) and, correspondingly, strength characteristics $\theta _i$ (Fig. 2D). Figure 2B and 2D show three curves, and they correspond to the psychometric function for a high-performing individual (16th percentile), a median-performing individual (50th percentile), and a low-performing individual (84th percentile). In the simulation, the true values of $\theta _i$ were distributed as $\theta _i \sim \text{ Normal }(3.25,.25^2)$ and the true value of $\beta$ was 2.0.

**Fig. 3:**
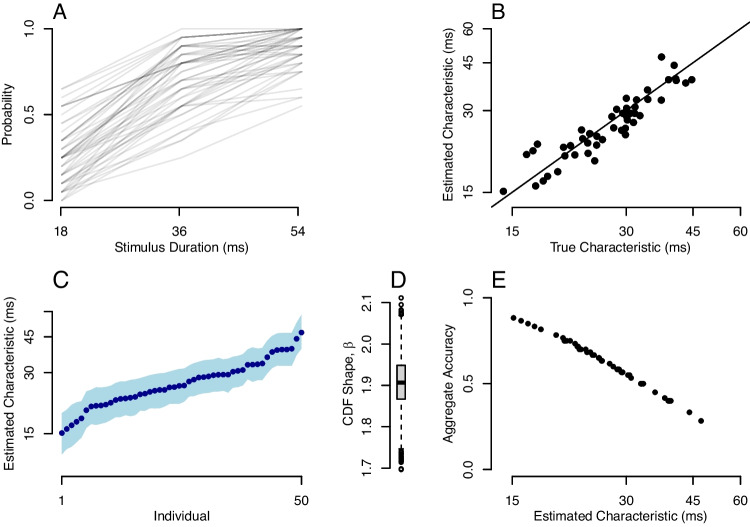
Application I. **A** Observed accuracy (probability) for 50 individuals across three durations in a hypothetical backwards-masking task with 20 trials per individual per duration. **B** Posterior mean of individual strength characteristic parameter as a function of true value shows the model performs well even with relatively few trials per individual. **C** Posterior distributions of individual parameters are constrained, resulting in a good ability to discriminate among individuals. The *points* are posterior means; the *shaded region* comprises the 95% credible intervals. **D** Posterior distribution of $\beta$, the shape of the psychometric functions is well localized. **E** Model parameters follow overall accuracy

Next, suppose the researcher gathers data on computer systems that have a 165-Hz refresh rate. This high refresh rate may be achieved with modern gaming monitors costing under $200 driven by the least expensive, integrated graphics cards. At 165 Hz, stimulus durations occur in increments of 6.06 ms, which is the *grain size* adopted here. The simulated experiment is comprised of 60 trials per individual which are divided equally into durations of 18.18 ms (three frames), 36.36 ms (six frames), and 54.54 ms (nine frames). We assume for the sake of convenience that the individual understands the task and is sufficiently practiced so that performance is stable across the durations. Observed accuracies across the 50 individuals are shown in Fig. 3A. There is a lot of diagnostic information evident in the data. The increase in accuracy with duration is apparent, as is the ordering of individuals.

The data were analyzed using the hierarchical model $\mathcal {M}_1$. Model estimates of the strength characteristics are plotted as a function of true values in Fig. 3B, and as can be seen, the estimates are well calibrated without excessive noise. Although strengths are plotted, the axes are transformed to the corresponding duration scale for convenience. Figure 3C shows how well each participant’s strength characteristic is located. The posterior means are ordered by individual and plotted as points. The shaded area is the 95% credible interval. The plot shows that even in this relatively small design, individual characteristics are precisely measured and individual differences are easily discerned. Figure 3D shows the posterior for CDF shape ($\beta$), and it centers the true value without excessive noise.

Figure 3E shows that the strength characteristic reflects overall accuracy. The overall accuracy for each individual is simply the arithmetic mean of accuracy across the three durations. The model, in effect, provides a theoretical account of overall accuracy, which is reflected as an intensity or strength characteristic. This pattern is good news—it would have been quite disconcerting if these characteristics were not a monotonic function of overall accuracy.

### Application II: Synthetic data with an adaptive staircase

Many psychophysical experiments use an adaptive-staircase design. Perhaps the most common of these is the 2-down/1-up (2D1U) staircase (Levitt, 1971). In the 2D1U staircase, intensity is lowered after two correct responses and raised after one incorrect response. In the 2D1U staircase, in the long run, the intensity oscillates around a fixed threshold value corresponding to an accuracy of $\sqrt{2}/2$, which evaluates to approximately 0.707. The goal then is to estimate this intensity threshold for each individual.

Application II is a simulation of the 2D1U staircase using hierarchical model analysis. True values across 50 individuals were distributed as the same in Application I. On each trial, the probability of a correct response followed a lognormal psychometric function. Durations were adjusted in increments of 6.06 ms, the grain size for a 165-Hz refresh rate. In the simulation, there were 50 trials per individual. The probability of a correct response was determined by the stable-state distribution; there was no learning or fatigue. Nonetheless, let’s consider the first 20 trials nonstationary and analyze the last 30 trials per individual.

**Fig. 4:**
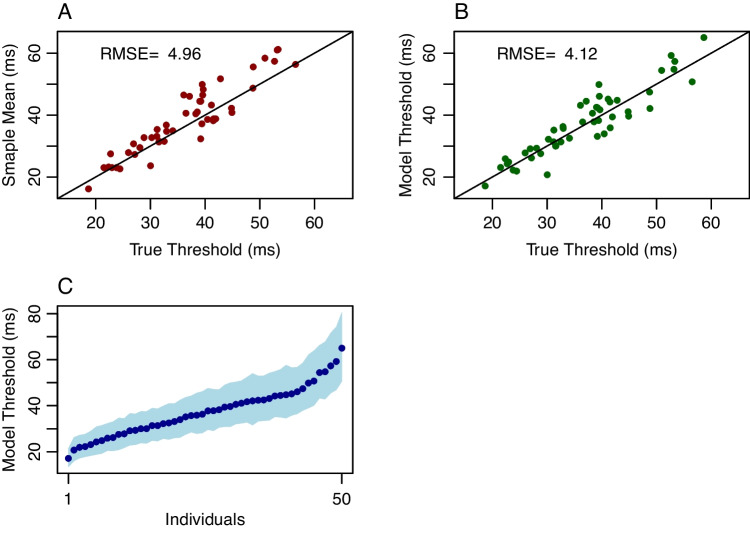
Application II. Data are generated with a 2-Down/1-Up staircase. **A** Conventional threshold estimates are sample means of duration across 30 steady-state trials. These are shown as a function of true thresholds $\eta _i$ and the RMSE miss is about 4.96 ms. **B** Model posterior means as a function of true values for the 30 steady-state trials; the RMSE miss is about 4.12 ms. **C** Model threshold posterior means of $\eta _i$ (*points*) and credible intervals (*shaded area*) show good localization even with 30 trials

In simulations, we specify a true intensity characteristic $\tau _i$. Under the lognormal model, $\tau _i$ is the intensity at which accuracy is 0.5; it is not the intensity corresponding to the 2-down/1-up target accuracy $\sqrt{2}/2 \approx 0.707$. Let $\eta _i$ denote the true intensity threshold at this criterion. It is straightforward to show that $\eta _i = K \tau _i$, where $K = \exp {\Phi ^{-1}(\sqrt{2}/2)/2} \approx 1.3$ is a constant. Taking $\eta _i$ as the true threshold at 0.707 accuracy, we can now compare conventional and model-based estimates of this quantity.

**Fig. 5:**
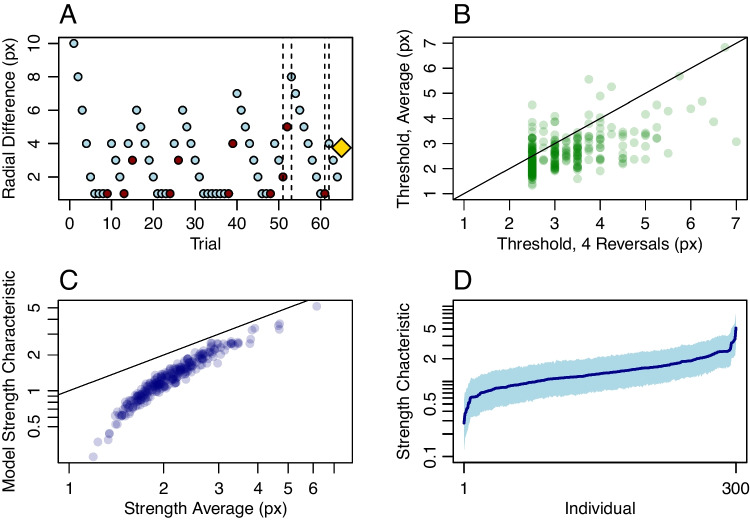
Application III. Circle-area discrimination thresholds in the Tsukahara et al. (2020) data set. **A** Example weighted 1-up/1-down staircase for a single participant; *vertical lines* mark the last four reversals and the *diamond* marks the four-reversal threshold estimate from the original authors. **B** Four-reversal thresholds plotted against sample-mean thresholds show a lack of agreement. **C** Threshold estimates from the hierarchical model (posterior means) plotted against sample means. The hierarchical model corrects for minimal intensity values in the chain. **D** Individual hierarchical-model threshold estimates with 95% credible intervals. Individuals are ordered from best (smallest threshold) to worst (largest threshold)

The conventional approach is to estimate the threshold from each individual’s duration data alone. Here, one can average reversal durations, or, more easily, average durations across the 30 included trials per individual. These values as a function of true thresholds $\eta _i$ are shown in Fig. 4A. The average miss, the *root mean square error* (RMSE), is also shown, and it is 4.96 ms. Such a value is surprisingly precise given the small number of trials. Of course, the situation is idealized; there is no learning, fatigue, attention lapses, or other factors that affect real-world performance.

The hierarchical model was analyzed with a free shape parameter $\beta$ as before. Yet, with such a small amount of data and a procedure designed to keep probabilities in a narrow range, there is an unfortunate complication. There is a multiplicative trade-off between $\beta$ and $\theta$, whereas one could preserve the probability while multiplying $\beta$ by a constant and dividing $\theta$ by the same constant. This trade-off resolves if there are several well-spaced durations or over 100 individuals, but it does not here in the adaptive setup. We followed what is often done in the psychophysical literature—we modeled individuals as coming from a one-parameter family by fixing $\beta$ to a known value (Green, 1990; Watson & Pelli, 1983).

The model results are shown in Fig. 4B. Here, there is even a greater degree of precision as the RMSE is 4.12 ms. Figure 4C shows the posterior distribution of threshold $\eta _i$. Even with as few as 30 trials per individual, individuals are functionally distinct and the range of variation is greater than the uncertainty in threshold estimation.

### Application III. Real-world example of a staircase

To show the usefulness of the approach, we reanalyzed the sensory discrimination data from Tsukahara et al. (2020). The researchers used four sensory discrimination tasks to study the relations among perception, attentional control, working memory, and fluid intelligence. The four sensory discrimination tasks, our target in this paper, are visual area discrimination, visual line-length discrimination, auditory pitch discrimination, and auditory loudness discrimination. To understand how the model estimates individual thresholds, we reanalyze the visual-area discrimination task. In this task, individuals were presented with two circles and had to judge which one was larger.

The Tsukahara et al. data set is an informative example of how researchers use psychophysical tasks to explore the influence of perception on other elements of mental life. Here, 300 individuals performed 64 trials in each sensory discrimination task. Figure 5A shows the procedure for a typical individual. The staircase was a weighted 1U1D procedure with the upward increment being three times the downward increment. The asymptotic accuracy of this procedure is 0.75, that is, the estimated thresholds are intensities needed for 0.75 accuracy.

From a psychophysical point-of-view, this is a difficult case because: (i) there are few trials per individual; (ii) the grain size is large (iii) there are several observations that are at the minimum of 1 pixel difference. Yet, in large batteries where individuals run a dozen or more tasks, it is often necessary to limit the number of trials per task. Moreover, staircase designs in these batteries often have large grain sizes, which are problematic for conventional threshold estimation. To foreshadow, the hierarchical models are exceedingly useful in these cases.

Tsukahara et al. estimated thresholds by averaging the intensity (radial difference in pixels) at the last four reversals. The last four reversals are shown as vertical lines, and the average is the diamond-shaped point. This estimate is biased high because two of the reversal points are error responses followed by correct responses, reflecting the influence of large upward steps. An alternative estimate is to take the sample of so many trials after a warm-up. We took the warm-up to be ten trials and averaged the 54 remaining ones. A plot of the Tsukahara et al. estimates vs. this sample-mean alternative is shown in Fig. 5B. Not only is the estimate higher in the reversal scores, there is also little correlation between the estimates across the two threshold estimators. The sample mean estimate is also biased high, though not as high as the last-four-reversals estimate. The problem is that for many individuals, intensities reach a minimal value of 1 pixel of difference. When individuals respond correctly, the staircase cannot go lower in intensity, though the performance warrants it.

We fit the hierarchical model to the staircases. Here, the difference served as an intensity parameter, and the lognormal psychometric function was employed. We transformed the strength characteristics to intensities and plotted the results as a function of the sample-mean estimates (see Fig. 5C). In contrast to the sample mean method, the model is robust to lower minimum intensities. Correct responses here serve as evidence for lower thresholds and estimated thresholds may even be below the minimum intensity. The model accounts for difficulties in the design that affect more conventional nonparametric methods.

Figure 5D shows the uncertainty in each individual’s threshold estimate as 95% credible intervals. These estimates are fairly well localized—it is obvious which individuals have relatively high and low circle-area discrimination. Hence, it should be possible to correlate this ability with other abilities, as is done subsequently.

## The correlation between two tasks

Consider the problem of studying the relationship between two psychophysical tasks, for example, whether people with shorter-duration thresholds in visual backward masking can better discriminate a faint tone in noise in a hearing task.

### Specification

The model is as follows: Let $Y_{ijk}=0,1$ be the performance for the $i$th individual ($i=1,\ldots ,I$) in the $j$th task ($j=1,2$) for the $k$th trial ($k=1,\ldots ,K_{ij}$).

The data level is a straightforward expansion of the previous model.

$$ Y_{ijk} \mid \theta _{ij} \sim \text{ Bernoulli }[p^*(s_{ijk},\theta _{ij},\beta _j)]. $$

Here, there is a separate true strength, $\theta _{ij}$, for each individual on each task, and there is a separate shape parameter $\beta _j$ for each task.

The latent model describes how $\theta _{ij}$ varies across individuals and tasks. Matrix notation proves convenient for specification. Let

$$ \boldsymbol{\theta }_i = \begin{pmatrix} \theta _{i1}\\ \theta _{i2} \end{pmatrix},\quad \boldsymbol{\mu } = \begin{pmatrix} \mu _{1}\\ \mu _{2} \end{pmatrix}, \quad \boldsymbol{\Sigma } = \begin{pmatrix} \sigma ^2_{1} & \rho \sigma _1\sigma _2\\ \rho \sigma _1\sigma _2 & \sigma ^2_{2} \end{pmatrix}, $$

be a vector of individual true strength characteristics, a vector of population strength characteristics, and a covariance matrix describing population variance and correlation across the tasks. Then, the latent model is

$$ \boldsymbol{\theta }_i \sim \mathrm{ N}_2(\boldsymbol{\mu },\boldsymbol{\Sigma }), $$

where $\text{ N}_2$ denotes a bivariate normal distribution.

In applications, we fit the following lognormal bivariate model:

$$ \begin{aligned} \mathcal {M}_2: \quad Y_{ijk} \mid \theta _i&\sim \text{ Bernoulli }(p_{ijk}),\\ p_{ijk}&= \Phi \left[ \beta _j\left( \frac{d_{ijk}}{d_{r_j}}-\theta _{ij}\right) \right] ,\\ \boldsymbol{\theta }_i&\sim \mathrm{ N}_2(\boldsymbol{\mu },\boldsymbol{\Sigma }). \end{aligned} $$

**Fig. 6:**
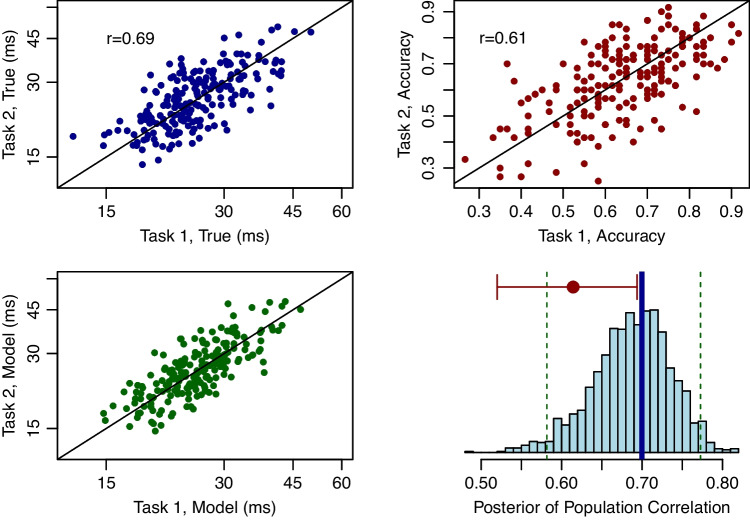
Application IV, correlations across tasks for synthetic data. **A** Scatter plot among true individual strength characteristics. The correlation among these 200 is close to the population value of $\rho =.7$. **B** Scatter of observed accuracy shows some attenuation. **C** Scatter of posterior means from the model shows shrinkage to a regression line. **D** Posterior distribution of population correlation $\rho$. This distribution is well centered around the true value (shown with a *thicker vertical line*). The *dashed lines* are credible intervals showing the degree of localization. The *red dot* toward the top of the plot is the observed correlation, and the *error bar* around it is the 95% confidence interval. This CI excludes the true value, showing overconfidence in an attenuated value

### Analysis

Analysis proceeds fairly similarly to before. Normal priors are appropriate for $\mu _1$ and $\mu _2$ and a gamma prior is appropriate for $\beta$. The remaining prior is on the variance matrix $\boldsymbol{\Sigma }$. There are several approaches to placing priors on a variance matrix. One approach is to place a single multivariate prior across all elements of $\boldsymbol{\Sigma }$, and examples of this approach are the inverse Wishart distribution (O’Hagan & Forster, 2004) and scaled inverse Wishart distribution (Huang & Wand, 2013). The other choice is to place separate priors on variance and correlation parameters, and a good example of this approach is the LKJ prior (Lewandowski et al., 2009). We have used both of these choices in various applications. Yang and Rouder (2025) provides a study of all three priors in hierarchical contexts, and based on that work, we chose the scaled inverse Wishart because it does not impart much prior information and is computationally convenient.

### Application IV: Synthetic backwards masking

Does performance on one backwards masking task correlate with performance on another backwards masking task? We constructed a synthetic example where 200 individuals performed 60 trials in each of two tasks. The design was the method constant stimuli for both tasks. Three duration levels, 18 ms, 36, ms, and 55 ms, were used in the backwards masking task, with 20 trials at each level (same as in Application 1). The true backwards-masking strength characteristics and shapes within each task were distributed as in Application 1.

Across tasks, true strength characteristics were correlated at a .70 value in the population. The true strengths for the 200 individuals are shown in Fig. 6A. The next column, Figure 6B, shows the correlation across observed accuracy (60 trials per individual). Here, the correlation, .61, is attenuated from the finite numbers of trials. This attenuation is not present in the hierarchical model estimates. The estimates (posterior means) of individual strength characteristics are shown in Fig. 6C. We do not recommend taking the sample correlation of these posterior means. That correlation treats the posterior means as if they were observed without variation and, in general, will not equal the posterior mean of the population correlation. The appropriate approach is to compute a posterior distribution of correlation values. Fortunately, the model includes a population-level correlation parameter, $\rho$, which may be used to generalize to new people. The posterior distribution of $\rho$ is shown in Fig. 6D. The true value—the target of inference—is .7. This value is indicated with a thicker solid vertical line. The posterior credible intervals, which denote a range of uncertainty, are indicated with dashed vertical lines. The true value is well within this 95% interval of uncertainty, indicating good calibration. This good calibration is in contrast with the poor calibration of the sample correlation from the sample accuracies in Fig. 6B. The upper red horizontal line corresponds to the sample correlation. The large red dot is the observed value of .61. The width of the red line shows the 95% confidence interval on this sample correlation. Here, the sample correlation is so attenuated that the CI does not cover the true value of 0.7.

In summary, with hierarchical models, the correlations across tasks may be estimated without attenuation. Moreover, the uncertainty in these correlations is readily attainable, and this uncertainty reflects variation from a finite number of trials and a finite number of people (Rouder & Mehrvarz, 2024).

### Application V: Correlation between circle and line length discrimination

Tsukahara et al. (2020) ran four sensory discrimination tasks as part of their study of the relations between sensory abilities, attentional control, working memory, and fluid intelligence. Here, we use a bivariate hierarchical model to explore the correlation between visual area discrimination and vertical line length discrimination in their data.

**Fig. 7:**
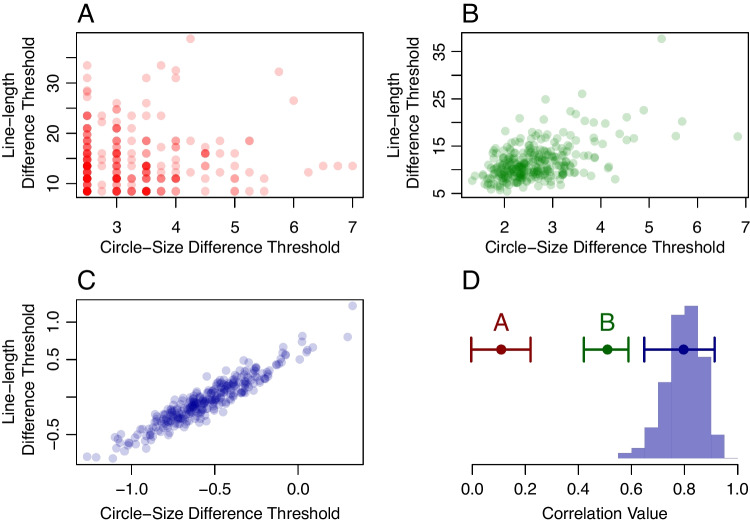
Application V, correlation between circle-area and line-length thresholds in the Tsukahara et al. (2020) data set. **A** Thresholds from the last-four-reversals estimates used in Tsukahara et al. show weak correlation. **B** Thresholds from sample-mean estimates show a moderate correlation. **C** Posterior means of individual thresholds $\theta _{ij}$ from the bivariate hierarchical model show a strong correlation. **D** Posterior distribution of the population correlation from the hierarchical model. The *horizontal bars* labeled A and B indicate 95% confidence intervals for the conventional correlations based on last-four-reversal and sample-mean thresholds, respectively. The *unlabeled horizontal bar* is the 95% credible interval from the model

In the battery, 300 participants ran 64 staircased trials in a circle-area discrimination task and a line-length discrimination task. The staircase was a 1U1D design with upward increments three times the size of downward increments. Tsukahara et al. averaged the last four reversals as a threshold estimate, and the scatter of thresholds across the two tasks is shown in Fig. 7A. The correlation, 0.11, is smaller than we might anticipate given that both tasks rely on visual acuity. An alternative sample approach to estimating thresholds is to take the sample mean across the intensity values after a 10-trial learning period. Figure 7B shows the scatter for these sample-mean threshold estimates. The correlation is far more pronounced, confirming that the last-four-reversal estimates are highly noise-prone.

Sample-mean estimates, while less noisy than the last-four-reversal estimates, are still variable due to the limited number of trials per individual per task. This variability may attenuate the correlation. To disattenuate the correlation, we analyzed the staircases for these two tasks with the bivariate hierarchical model. The resulting scatter plot of posterior means for $\theta _{ij}$ is shown in Fig. 7C. Accordingly, while the sample means were a significant improvement over the last-four-reversal estimates, they still reflected substantial trial noise, which reduced the estimate of correlation. The posterior of the population correlation is shown as a histogram in Fig. 7D. The 95% confidence intervals are provided for the last-four-reversal correlation estimates (denoted “A”) and for the sample mean correlation estimates (denoted “B”). These are far lower than the 95% credible intervals for the model-based correlation. In our judgment, these high model-based correlations are entirely plausible as both area discrimination and line-length discrimination are measures of a common visual acuity ability.

## Factor models

In the previous section, the correlation between two tasks was assessed. In contemporary individual-differences research, however, it is typical to administer a battery of several tasks. To accommodate this, the model is extended by specifying a latent-level structure, $\boldsymbol{\theta }_i \sim \mathrm{ N}_J(\boldsymbol{\mu }, \boldsymbol{\Sigma })$, where $J$ denotes the number of tasks, $\text{ N}_J$ is the $J$-dimensional multivariate normal distribution, $\boldsymbol{\mu }$ is the mean vector across tasks, and $\boldsymbol{\Sigma }$ is the $J \times J$ covariance matrix that encodes the relationships among tasks. This hierarchical formulation enables more accurate estimation of all pairwise task correlations as the resulting estimates are disattenuated and the associated uncertainty reflects both the number of trials per task and the sample size of individuals.

**Fig. 8:**
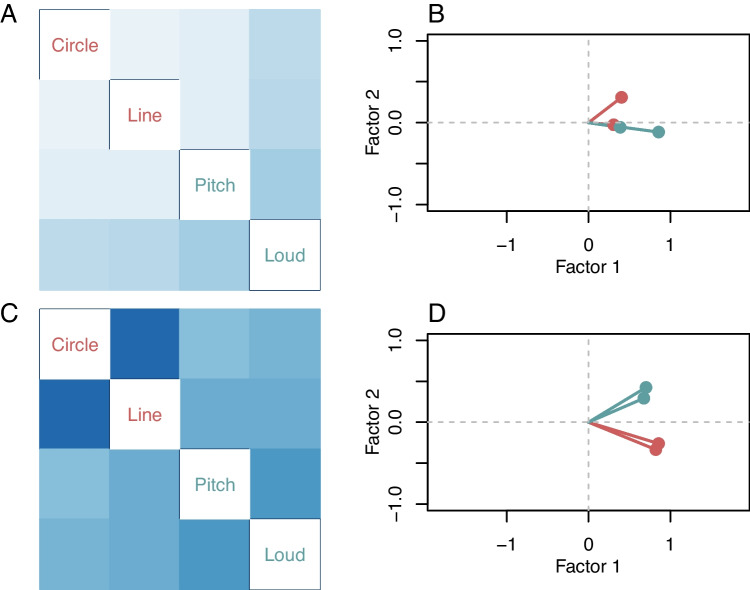
Application VI, relations among tasks in Tsukahara et al. (2020) data set. **A**-**B** Correlation matrix and factor loadings from the original authors’ last-four-reversals estimates. The correlations are modest, and the factor structure supports a one-factor interpretation. **C**-**D** Hierarchical factor model analysis. The correlations are more sizable, and there are separate factors for visual and auditory abilities

A central goal in individual-differences research is to identify the core dimensions that account for variability across tasks. Factor models provide a principled framework for this purpose. To illustrate the application of factor models in psychophysics, we revisit the data set of Tsukahara et al. (2020). In this study, individuals completed four tasks: two visual acuity tasks, as previously analyzed, and two auditory discrimination tasks (pitch and loudness). Conventional factor analysis begins with a score matrix, where each entry represents a threshold estimate for a given individual on a given task. For the Tsukahara et al. data, this matrix comprises 300 individuals (rows) and four tasks (columns). Next, the correlations across tasks are estimated by computing the sample correlations across the columns of the score matrix. Following the original analysis, we use the last-four reversals in each staircase to compute these scores. The resulting correlation matrix, shown in Fig. 8A, summarizes the relationships among tasks.

Inspection of Fig. 8A indicates that these scores are largely consistent with a single underlying factor. The two auditory tasks (pitch and loudness) exhibit moderate correlations with each other and with the visual tasks, while the two visual tasks (circle and line) display weaker correlations both with each other and with the auditory tasks. To quantify this structure, we fit a factor model to the score matrix. The resulting factor loadings, shown in Fig. 8B, align along a single dominant dimension, with the auditory tasks exhibiting higher loadings than the visual tasks. The first factor explains 29.40% of the total variance, whereas the second factor accounts for only 6.20%. The data are essentially one-dimensional; moreover, auditory tasks load better on sensory discrimination than visual acuity tasks. Unfortunately, as will be shown, these conclusions are wrong as they reflect excessive noise in the last-four-reversal estimates.

The previous section demonstrated that the hierarchical model substantially disattenuates correlations. For instance, the estimated correlation between circle-area and line-length discrimination increased from 0.11, as obtained from last-four-reversals estimates, to 0.79 under the hierarchical model. Building on this result, we now extend the hierarchical approach to all four tasks in order to recover the underlying factor structure within a unified model.

A hierarchical psychophysics model with factor structure is as follows: Let $Y_{ijk}$ denote the response for the $i$th individual in the $j$th task on the $k$th trial. The data model is the same as previous: $Y_{ijk} \mid \theta _{ij} \sim \text{ Bernoulli }[p^*(s_{ijk},\theta _{ij},\beta _j)]$; the main change is in the latent model. A factor model is placed on $\theta _{ij}$:

1 $$\begin{aligned} \theta _{ij} \mid \phi _{i1},\ldots ,\phi _{iM} \sim \text{ N }\left( \nu _j+\sum _{m=1}^M \lambda _{jm}\phi _{im},\;\sigma _j^2\right) , \end{aligned}$$

where $M$ is the number of retained factors, $\lambda _{jm}$ is the factor loading for the $j$th task on the $m$th factor ($m=1,\ldots ,M$) and $\phi _{im}$ is the factor score for the $i$th person on the $m$th factor. There is a multiplicative nonidentifiability: multiplying the factor loadings by a constant and dividing the factor scores by the same constant does not affect the model. To account for this nonidentifiability, factor scores may have a fixed location and scale: $\phi _{im} \sim \text{ N }(0,1)$. With this constraint, it is possible to marginalize across factor scores, resulting in the following multivariate model:

2 $$\begin{aligned} \boldsymbol{\theta } \sim \mathrm{ N}_J(\boldsymbol{\nu },\;\boldsymbol{\lambda }\boldsymbol{\lambda }'+D(\boldsymbol{\sigma }^2)), \end{aligned}$$

where $\boldsymbol{\nu }=(\nu _1,\ldots ,\nu _J)'$ is a vector of means, $\boldsymbol{\lambda }$ is a $J\times M$ matrix of loadings, and $D(\boldsymbol{\Sigma }^2)$ is a diagonal matrix with $\boldsymbol{\sigma }^2=(\sigma _1^2,\ldots ,\sigma _J^2)'$, a vector of variances, on the diagonal.

The above model is more complicated than conventional factor models. In conventional models, there is a single score per person per task. Here, there are a great many observations per person per task, and these are often at different strengths or intensities. Analysis proceeds through the Bayesian framework. Bayesian analysis of the factor model has a rich history (Ando, 2009; Bhattacharya & Dunson, 2011; Ghosh & Dunson, 2009; Lopes & West, 2004; Martin & McDonald, 1975; Merkle et al., 2021; Papastamoulis & Ntzoufras, 2022; Ročková & George, 2016). The above model is an example of a hierarchical factor model, and hierarchical factor models have recently been developed by Rouder et al. (2024), Stevenson et al. (2024), and Mehrvarz and Rouder (2025).

### Application VI

To illustrate the hierarchical factor model, we fit a two-factor version. The key parameters are the factor loadings $\boldsymbol{\lambda }$ and the covariance matrix $\boldsymbol{\Omega } = \boldsymbol{\lambda }\boldsymbol{\lambda }' + D(\boldsymbol{\sigma }^2)$. The associated correlation matrix is derived from $\boldsymbol{\Omega }$ and is presented in Fig. 8C. The corresponding factor loadings, shown in Fig. 8D, indicate the presence of two factors: a general sensory discrimination factor, which explains 59.50% of the variance, and a second factor distinguishing auditory from visual tasks, which explains 13.10% of the variance. This hierarchical approach yields a disattenuated and interpretable account of the relationships among the tasks.

## Thresholds for unconscious processing

Since Freud, researchers have been exploring the existence and boundary conditions of unconscious processing (Greenwald, 1992). Many modern studies take a two-task approach where researchers demonstrate that a *prime* that cannot be identified or detected nonetheless affects subsequent processing of a *target* (e.g., Vorberg et al., 2003). Demonstrating that a prime affects a target is straightforward, and statistically assessing the significance of such an effect may be done with run-of-the-mill methods. Demonstrating that a prime is undetectable is far more difficult on several accounts (Rouder et al., 2007). First, there is the thorny problem of supporting the null hypothesis of no detection (Morey et al., 2018). Second, there is the equally thorny issue of individual variation. The usual course is to assess whether the mean of detection across a collection of individuals is reasonably close to an appropriate at-chance floor value. Yet, it is highly likely that people differ markedly, and this variation complicates the interpretation of tests of means (Pratte & Rouder, 2009).

**Fig. 9:**
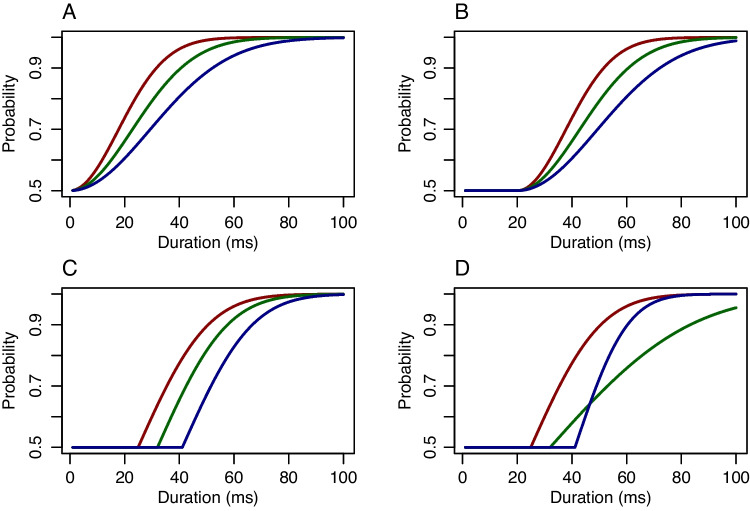
Various approaches to restricting the range of probability and modeling at-chance thresholds. **A** Linear compression of the range to [.5,1] interval. There is no intensity where performance is at chance. **B** A shift in the psychometric function provides for an at-chance threshold. All individuals have the same at-chance threshold at 20 ms. **C** Varying at-chance thresholds and a common intensity characteristic. **D** A more complex model with varying at-chance thresholds and varying intensity characteristics may violate stochastic dominance. **A**–**C** show setups that may be too simple; panel **D** shows one that may be too complex

What is the relationship between psychophysics and consciousness? Psychophysics was motivated in part by a metaphysical problem proposed by Leibniz in the late 18th century (Boring, 1950). Morey and Rouder characterized the problem as follows: “Leibniz noted that one may be conscious of the sound of a wave at a beach but that the sound of each drop of water crashing remains unnoticed. Given that the wave is nothing more than the sum of droplets, how could the sum of unnoticed elements reach consciousness? To Leibniz, each droplet left a *petite perception* that was more intense than the absence of a droplet but not sufficient in itself for consciousness. Because these petite perceptions have finite intensity, they may sum past a threshold level, that is, their sum may be consciously perceived.” (Rouder & Morey, 2009, p. 656)

Fechner (1860) codified the threshold as the intensity that divided consciousness from unconsciousness. He wrote:

The following phenomenon is inherent in the nature of the threshold. The farther the stimulus or stimulus difference falls below the threshold, the less chance there is for the stimulus or stimulus difference to be perceived and the greater must be the amount added before sensation can be felt. As long as the stimulus or stimulus difference remains below threshold, its perception is, as one says, unconscious. (pp. 205–206)

Although the above passage seems remarkably modern, given that it is over 150 years old, it is not representative of modern psychophysics. In the modern formulation, the threshold does not divide consciousness from unconsciousness. Instead, it corresponds to a criterial level of performance somewhere between floor and ceiling. For example, if one adopts a two-down one-up staircase, performance at threshold is guaranteed to converge to an accuracy rate of $\sqrt{2}/2$. The value is one of statistical convenience, and we are unaware of anyone who asserts it cleaves consciousness from unconsciousness. In modern psychophysics, the goal is to study how changes in the threshold across conditions follow lawful patterns and implicate certain processing architectures (e.g., Falmagne, 1990). Even though this goal is laudable and attractive, nonetheless, consciousness has been abstracted away. Yet, consciousness remains an important theoretical concern, especially in fields adjacent to psychophysics.

The natural value that does cleave consciousness from unconsciousness is the highest stimulus value that corresponds to a chance level of performance. In a two-choice case, for example, at-chance accuracy is naturally defined at $p = .5$. The goal, then, is to find the highest intensity that divides accuracy at 0.5 from accuracy above 0.5. We call these intensities the *at-chance thresholds*. The following describes how chance levels are typically incorporated into psychophysical curves and what new adaptations are needed to measure at-chance thresholds.

### Forced choice and compressed probability

The typical approach to modeling above zero levels of at-chance performance is to compress the probability range (e.g., King-Smith et al., 1994). Psychometric curves may be compressed to the range $[p_0,p_1]$ by linear scaling:

3 $$\begin{aligned} G(x,\ldots )= p_0+(p_1-p_0)F(x,\ldots ), \end{aligned}$$

where $F(x,\ldots )$ is a psychometric function on either intensity of strength and $G(x,\ldots )$ is the corresponding compressed psychometric curve. Figure 9A shows an example on intensity where $p_0 = .5$ and $p_1 = 1$; the curves are for three hypothetical people. The curves are those for Fig. 2A compressed to the range for two-alternative forced choice.

### Thresholds on compressed probabilities

Compression does not address the problem of at-chance thresholds. Indeed, all positive intensities correspond to above-floor performance. The most natural way to capture an at-chance threshold is to shift the curves. Figure 9B provides examples with an at-chance-threshold at 20 ms. After 20 ms, the probability rises above the floor of $p_0 = 0.5$.

**Fig. 10:**
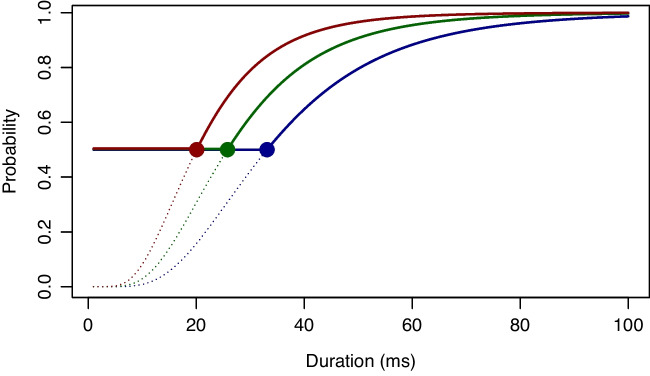
A truncation approach to at-chance thresholds. Each individual has a single intensity characteristic parameter. The truncation at 0.5 provides for a natural at-chance threshold that is a function of the intensity characteristic

Figure 9B shows a case where three individuals have varying intensity characteristics but a common threshold. This setup is constraining—it is reasonable to expect individual variation in threshold and unreasonable to expect a lack of such variation. Rouder et al. (2007) was perhaps the first to use a hierarchical model to assess thresholds in individuals, and their psychometric functions—based on a half normal—are shown in Fig. 9C. Rouder et al. (2007) allowed each individual to have their own threshold, but there was a common intensity characteristic. The result was that psychometric functions across individuals were shifted from one another on the intensity scale. In testing this setup, Morey et al. (2008) found a noticeable violation in backwards masking—people who needed more time for performance to be above floor also had a shallower psychometric curve. To account for this phenomenon, Morey et al. (2009) developed psychometric curves and associated hierarchical models where each individual had their own threshold and intensity characteristic. Figure 9D shows three possible curves.

**Fig. 11:**
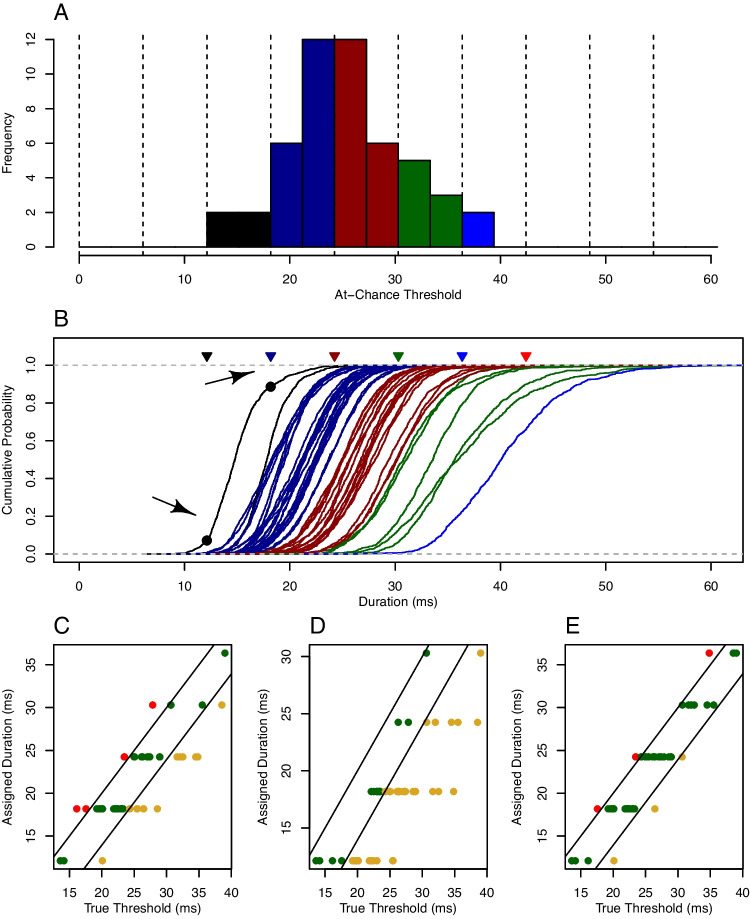
Application VII, at-chance thresholds. **A** True at-chance thresholds. The *colors* indicate the maximum available duration at 165 Hz for assessing subliminal priming. **B** Posterior CDFs of at-chance thresholds; the *colors* indicate maximum available duration based on a probability cut-off of 0.5 (see text for details). **C**-**E** Assigned duration as a function of true at-chance threshold for cut-offs at 0.5, and 0.1, and with a fivefold increase in trial size, respectively

All four examples in Fig. 9 are problematic for studying unconscious priming in psychophysical adjacent fields. Figure 9A contains no threshold; Figure 9B does not allow for individual differences in threshold; Figure 9C may be workable, but the constraint of invariant intensity characteristics seems difficult. Figure 9D may seem appropriate at first glance. Yet, we think it is too flexible and entails the following two problems: First, the model becomes difficult to analyze in small-trial-size settings with each individual described by two parameters, a threshold and an intensity characteristic. Using two parameters per individual will require much more data and may limit gains from hierarchical regularization. Second, the model is so flexible that it implies violations of a reasonable property called *stochastic dominance*. Stochastic dominance is the property that if Individual A truly performs better than Individual B at one intensity, then Individual A performs better than Individual B at all intensities within an ecologically valid range. All the curves in Fig. 9A-C obey stochastic dominance. Yet, two of the curves in Fig. 9D violate it as two curves cross. We are unaware of violations of stochastic dominance in any perceptual domain, and having models that allow it strikes us as unwise in a measurement context.

The setup we advocate is shown in Fig. 10. The main feature is the use of a maximum operator rather than a compression. The psychometric function is:

4 $$\begin{aligned} \text{ At-Chance } \text{ Threshold }: \quad p(d,\tau _i,\beta )= p_0 \vee F_+\left( \frac{d}{\tau _i},\boldsymbol{\beta }\right) \, \end{aligned}$$

where $\vee$ is the maximum relation, $p_0$ is the floor, and $F_+$ is a CDF that ranges from 0 to 1 on positive intensities. The solid lines show the values of this psychometric function; the dashed lines show the parts of $F_+$ that are not observed because they are below the floor. The dot is the at-chance threshold—it is the largest intensity for which performance is still at chance. This setup has a few advantages: 1. There is a single parameter, $\tau _i$, that simultaneously determines the intensity characteristic and the threshold. Individuals with smaller thresholds have quicker rises in probability with intensity. 2. Stochastic dominance is guaranteed. Individuals with lower values of $\tau _i$ perform better than those with higher values for all intensities.

### Application VII

Subliminal priming occurs when primes that are undetectable still affect responses to the subsequent target. In many cases, the critical setting is the intensity of the prime. If it is set too high, then the prime will be detectable; if it is set too low, there may be no subsequent priming of the target. The goal then is to find the maximum prime intensity for undetectability so that that intensity may be used to assess priming. We show how the hierarchical model with at-floor thresholds may be used to find this intensity with a small number of trials per individual.

In the application truncated lognormal psychometric functions are placed on duration. In this parametric form, the value of $\tau$ corresponds exactly to the maximum duration at chance. Fifty synthetic individuals provided 60 prime-detection trials across three durations as in Application I. True values of $\tau _i$ were sampled as before, and a histogram of each individual’s at-chance threshold ($\tau$) is shown in Fig. 11A. If one knew these values and one could finely adjust the duration of the prime to arbitrary precision, then one could show primes for this duration in the priming task. Yet, we neither know these values nor can we set durations to arbitrary precision in the priming task. On modern monitors, there are a small number of discrete durations that may be used, that is, the grain size is large. The vertical dotted lines in Fig. 11A show the available duration values for a 165-Hz monitor. To show subliminal priming, the researcher should choose the maximum available duration less than the at-chance threshold. For example, if a true at-chance threshold was 15 ms for a particular individual, the largest available duration less than this value is 12.12 ms, or 2 refreshes at 165 Hz. The color of the bins indicates the largest available durations for given at-chance thresholds. These duration assignments are best-case and serve as a standard for assessing the hierarchical model’s performance.

The resulting trial data from these true values were analyzed, and the critical target is the posterior distributions of the at-chance threshold. These posteriors for each person are shown in Fig. 11B as cumulative distribution functions (CDFs). The value on the $x$-axis is a duration value $d$; the value on the *y*-axis is the probability that this duration results in above-chance performance. There is one curve for each individual, and curves to the left indicate better performance (higher probability of above-chance performance at set durations). Take, for example, the left-most curve for the best-performing individual. There are arrows highlighting points with durations of 12.12 ms and 18.18 ms. The corresponding probabilities are about 0.2 and 0.96, respectively, indicating that the probability the 12.12-ms duration is above the at-chance threshold for this individual is 0.2, and the probability the 18.18-ms duration is above the at-chance threshold is 0.96. These CDF curves may be used to make informed decisions about which durations are subliminal for each individual. The 18.18-ms duration is too slow for the highlighted individual, as the probability of superliminality is too high; the 12.12-ms duration is plausibly subliminal, as there is only a 20% chance of superliminality for this individual.

The colors of the lines in Fig. 11B show the duration assignment for subsequent priming experiments. The shown assignments were made at a $p = .5$ criterion, that is, the assignment is the largest available duration at 165 Hz so long as the probability of superliminality is less than 0.5. How good are these assignments compared to the assignments in Fig. 11A? There are three possible outcomes: First, the assignment can be *just right*. Just-right assignments are below the true at-chance threshold, but not more than a refresh interval (6.06 ms) below. Conservative assignments are more than a refresh interval below the true at-chance threshold. These conservative assignments are so far below the true value such that a longer duration could have been assigned without violating subliminality. The opposite problem is a liberal assignment. A liberal assignment is one above the true at-chance threshold. Figure 11C shows the assignments as a function of true at-chance threshold for each individual. The solid line is the diagonal; hence, subliminality occurs for points (individuals) below this diagonal. Liberal assignments are for points above this diagonal and are colored in red. The dashed line separates assignments more than a refresh interval below the true at-chance threshold, and those points below this dashed line correspond to assignments that are too conservative (yellow points). The points in between these lines, colored in green, correspond to just-right assignments. In the example, 0.62, 0.28, 0.10 of the assignments were just right, too conservative, and too liberal, respectively.

The choice of $p = .5$ tends to balance too liberal and too conservative assignments. Researchers may be interested in stressing conservative assignments at the expense of liberal ones. Lowering the value of $p$ stresses conservative assignments. Figure 11D shows the case for $p = .1$, and here the reduction of liberal assignments is offset by an increase in conservative assignments. Researchers who are unsatisfied with the prevalence of conservative assignments can improve the overall accuracy of assignments by increasing the number of trials per individual. In this example, individuals observe 20 trials at each of three durations or 60 trials overall. Figure 11E shows the case if the trial size is increased fivefold to a total of 300 trials overall ($p=.5$). Here, the proportion of just-right assignments is 0.86; the misses from the other assignments are marginal. In summary, hierarchical models with truncated psychometric functions may provide principled assessment of superliminal vs. subliminal performance.

## Model misspecification

Model misspecification refers to an accounting of how the model may miss the data. The models we provide are somewhat simpler than hierarchical psychometric models from Balestrucci et al. (2022), Prins (2024), and Schütt et al. (2016) as we have not included random slope parameters or random lapse parameters. We make this choice of simplicity for estimation accuracy and interpretability in small trial-size contexts, as is discussed subsequently. This advocacy of simplified models with only random shifts makes the issue of misspecification all the more important.

### Visualizing misspecification

In the model, it is possible to construct a posterior distribution of the probability for every observation. Observations, $Y$, are discrete, either in error or correct, and in the Tsukahara et al., there are about $N\approx 77,000$ such observations. For each of these 77,000 observations, the model makes a prediction in the form of $p_*$. Let $Y_n=0,1$ be the $n$th observation ($n=1,\ldots ,N$) and let $p^*_n$ be the corresponding probability, that is, the prediction for $Y_n$. It is possible to compute a posterior distribution for each $p^*_n$ and the posterior mean constitutes a fine-grained prediction useful in assessing misspecification. From these predictions, residuals may be calculated and plotted.

**Fig. 12:**
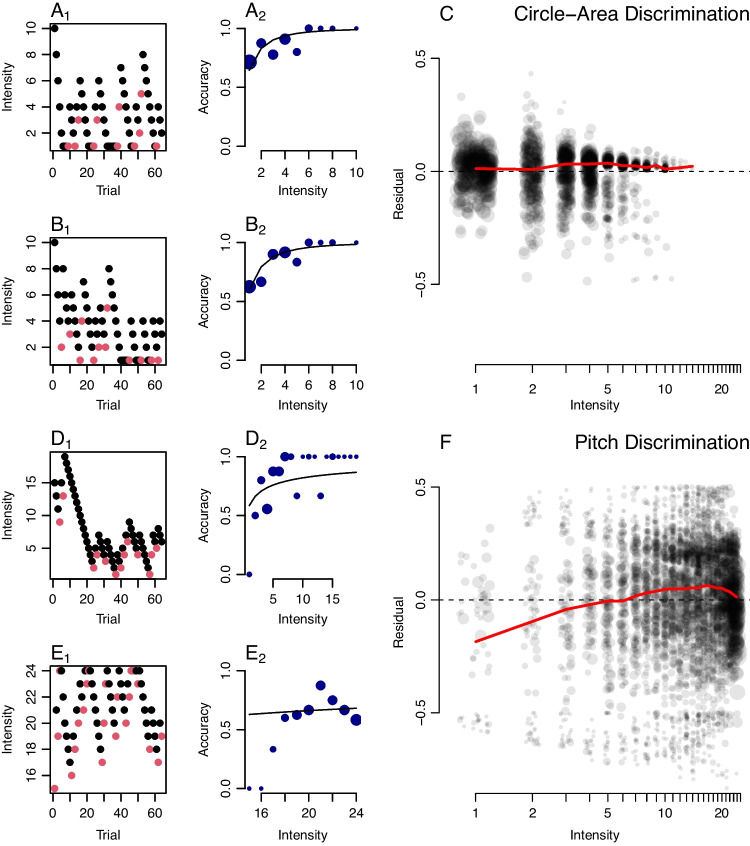
Assessment of model misspecification through posterior predictions for each observation. The model was fit to the Tsukahara et al. (2020) data set. **A**$_1$-**A**$_2$: Staircase for Subject 1 in circle-area discrimination and corresponding accuracy as a function of intensity. The *line* is model-predicted accuracy. **B**$_1$-**B**$_2$: The same for Subject 19 in the circle-area discrimination task. **C** Plot of residuals, the subtraction of predicted accuracy from observed accuracy, shows little evidence of misspecification in the circle-area discrimination task. **D**-**E** Staircase and accuracy plots for the same individuals in the pitch-discrimination task show difficult-to-interpret staircases and much misfit. **F** Residuals also reveal the misspecification for the pitch-discrimination task

Figure 12 shows visualizations of misspecification through these predictions. Figure 12A$_1$ shows the staircase for Subject 1 in Tsukahara et al.’s circle-area discrimination task. Figure 12A$_2$ shows the corresponding accuracy as a function of intensity. The observed values are points, and the point size indicates the number of trials at that intensity. The line shows the model’s predictions of accuracy, and for Subject 1 in circle-area discrimination, it is quite good. Panels B$_1$ and B$_2$ show the same for Subject 19 in the same task. Once again, the model predictions are reasonable. Indeed, these good-fit patterns hold generally for circle-area discrimination. Figure 12C shows the residuals—the predicted accuracy subtracted from the observed accuracy—as a function of intensity. The red line is a LOWESS smooth that captures overall trends. The residuals are fairly small and show no systematic pattern.

The remaining panels, Fig. 12D-F, show substantial misspecification. These panels are for Tsukahara et al.’s auditory pitch discrimination task. Figure 12D$_1$ and D$_2$ are for Subject 1 in the task, and the staircase shows some learning. The prediction, while well centered, is far from the points and misses low for larger intensities. Indeed, for many individuals, the predicted curve is too flat. Figure 12E$_1$ and E$_2$ show why—this curve is for Subject 19 in pitch discrimination. Here, the individual did so poorly that they were presented with the highest intensity pitch difference several times. The curve is flat because the individual does not improve with increasing intensity. There are individuals who show little-to-no improvement with increasing intensity, and as a result, predictions for all participants who share a common rate $\beta$ are fairly flat. Figure 12F shows the residuals, and they are problematic. The residuals are widely dispersed and exhibit systematic trends.

### Effects of misspecification

The misspecification in the pitch-discrimination task may reflect a problem with the task rather than the model. We suspect that some participants were still learning aspects of the task and had not reached steady-state behavior. These participants were certainly well motivated, as their staircases in the other tasks look reasonable. Instead, they had not yet learned the pitch ensemble and were relatively insensitive to pitch differences. Others, however, did learn the task and were far more sensitive. This difference among participants is reflected in different rates, a phenomenon the model does not capture. We think that rather than changing the model, it is better to change the task. Including a small amount of practice with feedback may have rectified the situation.

What is the effect of these misspecifications? The main goal in our reanalysis of Tsukahara is to capture the relations among the tasks. To understand the role of misspecification, we constructed a series of small simulations. The simulations are designed to mimic the misspecifications observed above. The first task is well-specified, that is, individuals follow a truncated psychometric function given by $p^*_{ij}=.5 \vee \Phi (\beta (s_{ij}-\theta _i))$. The second task is misspecified in some manner. For example, in one simulation, to mimic massive individual variation in slope, half of the individuals had a low ground-truth value of $\beta$, indicating poor understanding of the task, while the other half had a higher value, indicating more reasonable performance. There was a downward bias in correlation when the differences became extreme (a factor of 4 in $\beta$). Hence, the values we observe in Application VI, though much higher than the conventional estimates of correlations, may still be underestimated. Because the model is already greatly disattenuating conventional correlations (see Fig. 8), we are not too concerned with this participant element of misspecification.

### Causes and remedies for misspecification

#### Parametric psychometric functions

We use parametric forms such as the lognormal and Weibull on intensity (or normal and Gumbel on strength). We are not so much committed to any one parametric form, but the method is squarely parametric. An alternative is to avoid parametric assumptions altogether by relying on asymptotic behavior of staircases in the measurement of thresholds. This approach requires quite many trials and blocks, and for this reason, it is unattractive in psychophysically adjacent fields. We do not believe nonparametric approaches are useful in psychophysically adjacent domains where there are fewer observations per individual and collection is under less-than-ideal circumstances. For example, we showed in Application III that real-world data from Tsukahara are better modeled parametrically than with a nonparametric approach.

We think the most satisfying approach is to use a multiverse analysis. Researchers may modify the psychometric function by using a different distribution, for example, the Gumbel, logit, or the $t$ CDF. The question then is whether the pattern of correlations change as a result of the parametric form. The correlation values do depend modestly on the parametric form, and they decrease as the tails of the psychometric function become heavier. For example, correlations in the Tsukahara set are about 5% lower with a Gumbel psychometric function rather than a normal. The normal has thin tails, while the Gumbel and logit have heavy tails. Nonetheless, the pattern is the same.

#### Individuals vary in one way

We make the assumption that people vary in only one way. In the current modeling, individual psychometric curves on the strength are shifts or translations of one another. This random-shift property is seen clearly in the normal psychometric function for a task: $p^*_{ij}=\Phi (\beta (s_{ij}-\theta _i))$ where $i$ denotes the individual, $j$ denotes the trial, and $\theta _i$, the strength characteristic for the $i$th individual, describes the shift. The main advantage of this form is interpretability. Individuals are described with a single variable, and this compact form allows for principled inquiry of how individuals covary across tasks.

This assumption is likely violated in the pitch-discrimination task. When this assumption is violated, it seems natural to assign a different rate to each participant, e.g., $p^*_{ij}=\Phi (\beta _i(s_{ij}-\theta _i))$. This is a random random-intercept ($\beta _i$) and random-slope ($\theta _i$) model, and is implemented in Houpt and Bittner (2018) and Prins (2024) among others. We worry about three elements: I. Localizing two random parameters is difficult in 2AFC with small numbers of observations per individual because there is a pronounced ridge in the likelihood where smaller values of $\theta$ are associated with larger values of $\beta$. II. The model is so flexible that it violates stochastic dominance in which Person A can be better than Person B on low-intensity stimuli while Person B is better than Person A on high-intensity stimuli. III. Models with two individual-difference parameters require far more intensive modeling to understand variation across tasks, and the resulting analyses may be more difficult to interpret.

If one must generalize the random-shift model, we advocate retaining the theme that people vary in one way. Consider the following example for 2AFC where there is a natural lower bound at .5:

$$ p^*_{ij}= \frac{1}{2} \vee \Phi \left( \beta e^{-\theta _i}(s_{ij}-\theta _i)\right) $$

In this model, the rate and shift change together, ensuring stochastic dominance. Moreover, the estimation issues are alleviated, and the interpretation of $\theta _i$ as the individual’s strength remains intact. Other alternatives include individual-varying nuisance parameters such as lapse parameters (Houpt & Bittner, 2018), though we recommend doing so without random slopes. For example, rather than assuming an individualized rate, we may introduce an individual-specific, stimulus-invariant lapse parameter $\gamma _i$ such that $p^*_{ij}=\gamma _i/2+(1-\gamma _i)\Phi (\beta (s_{ij}-\theta _i))$. Here, lapses occur with probability $\gamma _i$ and if the participant lapses, they guess with a 50% chance of a correct response. These lapses serve as a nuisance and need not inform the relations among tasks, which are assessed by latent-variable models on random intercepts.

#### Conditional independence and dispersion

There is a conditional independence assumption where the probability of a correct response on the current trial is a function of the intensity on the current trial, the psychometric curve parameters, and nothing else. This conditional independence is a strong assumption that implies (i) steady-state performance and (ii) a lack of dependence among trials from variations in attention. The above visualizations are easily adapted to search for lack of steady-state behavior; one can plot the residuals as a function of trial number.

Perhaps, the more difficult case is overdispersion. Here, the probability of a correct response depends on the history as success may be breed an enhanced state where the participant is particularly attuned. The consequence of overdispersion may be significant—the analyst believes they have more information than there is in the data, and this belief leads to overconfidence in the assessment of thresholds and the structure governing them. Perhaps the best news here comes from our simulations. It seems that overdispersion needs to be quite large before there are noticeable effects on uncertainty estimates. For example, we ran a simulation where $\theta$ varied on each trial with a standard deviation of .25, which is just as large as how people vary in true values across our simulations. The effect on average correlations was not detectable; the effect on the credible intervals was slight.

There are revisions to models to account for overdispersion. Schütt et al. (2016) provide a beta-binomial approach to overdispersion that may be most applicable in this setting. The beta-binomial approach, however, does not capture the idea of enhanced attentional states that may persist for a number of trials. A more nuanced approach that allows for variation on set time scales is to place autoregressive models on strength characteristics. Examples of this approach in related domains include hierarchical diffusion models with time-varying parameters (Gunawan et al., 2022) and autoregressive lognormal race models (Rouder et al., 2015).

## Discussion

The key development here is a novel random-effects psychophysics for psychophysical-adjacent applications with small numbers of trials per individual per task. The approach licenses the study of individual differences in perception. With the hierarchical approach, differences across individuals are easily assessed, and correlations across tasks are accurately measured without attenuation. Moreover, the resulting measures of uncertainty reflect the number of trials per individual and the number of individuals. In applications that involve a large battery of tasks, it is possible to yoke thresholds to latent-variable models, such as factor and structural equation models. Even for researchers engaged in more traditional psychophysics with many trials and few participants, using the hierarchical approach to pool across participants improves the accuracy of threshold estimation. Finally, because the approach leverages the computational advantages of the Bayesian framework, the analyst is free to focus on creative model specifications custom-tailored for substantive problems. With this freedom, we propose at-chance threshold models custom-tailored for detecting subliminal stimulation.

The examples in this paper are based on a relatively small number of trials per individual per task. This small number seems strange compared to the thousands of trials typically required in most psychophysical studies. At first glance, it may seem that this small number is possible because of the pooling of information in the hierarchical model, but this pooling is only a small part of the story. The larger part of the story is that in psychophysical applications, people vary to a fairly large degree, and discriminating among them is not so difficult. In simulations, the standard deviation of strength is .25 and this corresponds to a standard deviation of 1.08 dB across people. On the intensity scale, there is a factor of 2 between the 10th and 90th percentile individuals in threshold. In our experience, this large degree of variation is warranted. For example, Morey et al. (2008) documents at-chance thresholds in backwards masking that vary by a factor of 3. In the Tsukahara et al. data used here, this difference is a factor of 1.9 or greater in all tasks. The main reason the approach works with such a small number of trials is this large variation among people. If researchers are more interested in within-subject contrasts across conditions, then individual variation is likely reduced, and the number of trials will need to be increased.

### Future directions

#### Stochastic dominance in individual differences in psychophysics

The models presented here prioritize a stochastic-dominance property within a domain. Stochastic dominance implies that if Person A responds more accurately than Person B for one intensity, Person A responds more accurately than Person B for all intensities. If this property holds in a domain, then it is reasonable to search for the one-way in which people differ, and the models proposed here are useful in this regard.

Stochastic dominance is called *item invariant ordering* in psychometrics (Sijtsma & Junker, 1996). To make the analogy, we consider each intensity level as an item, and its invariant ordering across individuals is the stochastic dominance property. In psychometrics, item-invariant ordering is used to select items. Good items obey stochastic dominance, and items that do not may be discarded (Sijtsma & van der Ark, 2017). Although there are several examples of researchers testing item invariant ordering (e.g., Myszkowski, 2020), we are unaware of tests of stochastic dominance across people and intensity levels in psychophysics. Such studies are sorely missing and would be a welcome addition.

#### Optimal design

We explored a few standard designs here, including the methods of constant stimuli and adaptive staircases. The adaptive staircase is useful when a single parameter of the psychometric function varies across people or conditions. It is less useful for the two-parameter psychometric functions used here, as the adaptive staircase may not cover a sufficiently large range of probabilities to estimate more than one parameter. It is for this reason that we demonstrated the model’s efficiency with the method of constant stimuli—even three levels are enough to accurately estimate individuals and uncover structure. Yet, there are more optimal schedules that can lead to better parameter recovery with fewer trials and individuals. The field of statistics for designing experiments to meet statistical criteria is called *optimal experimental design*, and advances have recently been developed in psychology by Myung and colleagues (Cavagnaro et al., 2010; Gu et al., 2016; Lesmes et al., 2015; Myung et al., 2013). Optimal design remains topical, and Kristensen et al. (2025) provides a detailed frequentist analysis of estimation in a task. They cover the complication that some parameters vary with individuals (e.g., $\theta _i$) while others do not (e.g., $\beta$). How to do optimal design calculations across a battery of tasks remains for future development.

### You can do it

We have entered a new and exciting phase in psychological statistics. In the past, one needed to be relatively an expert to analyze custom models like the ones presented here. Yet, with new tools, analysis of most of these models has become routine and straightforward.

The reason analysis has become so routine is the development of a flexible language, called BUGS (Lunn et al., 2000), and its implementation in packages such as Stan (Carpenter et al., 2017) and JAGS (Plummer, 2003). The genius of BUGS is that model specification is closely related to standard random-variable notation. For example, the random-variable specification $Y \sim \text{ Normal }(\mu ,\sigma ^2)$ is notated as Y~dnorm(mu,1/sigma2) in JAGS. With the development of these free, off-the-shelf tools, it is likely that if you can specify a model, you may be able to analyze it as well. In fact, you can input the random-variable specification into ChatGPT, and it will provide the JAGS or Stan code.

To help you implement these models, we have provided a R file, example.R at https://osf.io/sakt5/files/pvrjt. It is the R code behind Application I. The code is presented with commentary about the setup. Working through it should help provide an example of JAGS analysis. For those seeking more extensive examples, the working Rmarkdown version of this manuscript is available at the above URL. The code used in the analysis and drawing figures in this paper is included.

## Data Availability

NA

## Footnotes

1 The term *true* is used throughout to indicate a latent parameter. It stands in contrast to the term *sample* or *estimate*.

2 One way of highlighting the difficulty of taking logs of quantities with physical units is to take a Taylor expansion of the logarithm function: $\log (x) = (x-1) - \frac{1}{2}(x-1)^2+ \frac{1}{3}(x-1)^3 - \frac{1}{4}(x-1)^4 + \ldots$. Note that the sum is a pure number only if $x$ is a pure number. If $x$ has units, then the log is a vector of infinite dimension. For example, if $x$ is a length in feet, then $\log (x)$ is the sum of length $(x-1)$, area $\frac{1}{2}(x-1)^2$, volume $\frac{1}{3}(x-1)^3$, hypervolume $\frac{1}{4}(x-1)^4$ and so on. To avoid such uninterpretable outputs, logarithms are restricted to pure-number inputs.

## Code Availability

All files for typesetting this paper, performing the analyses, and drawing the figures are available at https://osf.io/sakt5/files/osfstorage (Version 2). Interested readers may start with the file p.Rmd to follow the development.
